# Salivary Duct Carcinoma with Rhabdoid Features of the Parotid Gland with No E-Cadherin Expression: A Report with Anti-HER2 Therapy and Review of the Literature

**DOI:** 10.3390/dj11100229

**Published:** 2023-09-25

**Authors:** Richa Jain, E. Ritter Sansoni, Jacqueline Angel, John P. Gleysteen, D. Neil Hayes, Adepitan A. Owosho

**Affiliations:** 1 Methodist Le Bonheur Healthcare, Memphis, TN 38104, USA; 2 Department of Otolaryngology, College of Medicine, The University of Tennessee Health Science Center, Memphis, TN 38103, USA; 3 Department of Radiology, College of Medicine, The University of Tennessee Health Science Center, Memphis, TN 38103, USA; 4 Department of Medicine, College of Medicine, The University of Tennessee Health Science Center, Memphis, TN 38103, USA; 5 Department of Diagnostic Sciences, College of Dentistry, The University of Tennessee Health Science Center, Memphis, TN 38103, USA

**Keywords:** E-cadherin loss, salivary duct carcinoma, salivary gland neoplasms, immunohistochemistry

## Abstract

Salivary duct carcinoma with rhabdoid features (SDC-RF) is a rare form of salivary gland neoplasm that was recently described. We report a case of SDC-RF of the parotid gland with loss of E-cadherin and decreased β-catenin expression in a 73-year-old male who presented with right facial/neck swelling and intermittent pain. Morphologically, the tumor presented with a discohesive infiltrate of isolated and cords of pleomorphic round cells containing moderate amount of eosinophilic to fine-vacuolated cytoplasm and hyperchromatic nuclei infiltrating through fibroadipose tissue and salivary parenchyma. Immunophenotypically, the tumor was positive for Cytokeratins Oscar and 7, GATA3, GCDFP, HER2, and an androgen receptor but negative for CK20, S100, p40, Melan A, CDX2, TTF1, ER, SATB2, DOG1, synaptophysin, and chromogranin. Due to its diffuse infiltrating pattern, involvement of the parapharyngeal space, supraclavicular fat pad, dermis, and skin without a defined surgical target, the tumor was deemed unresectable. Anti-HER2 therapy (Herceptin and Pertuzumab) was utilized. At the last follow-up, the patient is alive, with complete locoregional control and brain metastases. An electronic search was performed in the following registries for papers published up to June 2023: PubMed, Embase, and Web of Science. For the database searches, the keywords searched were “salivary gland”, “salivary duct carcinoma”, and “salivary duct carcinoma with rhabdoid features”. Our review of the literature identified 30 cases of SDC-RF that reveal there is a predilection for males (83%), parotid gland (72%), and patients older than the 6th decade of life (83%). Immunophenotypically, all SDC-RF cases except one were positive for AR and GCDFP (97%), 81% were positive for HER2, and loss or decreased expression of E-cadherin in 93% of cases. In conclusion, we described a rare case of SDF-RF of the parotid gland with no E-cadherin expression, decreased β-catenin expression, and its immunophenotypic profile.

## 1. Introduction

Salivary duct carcinoma (SDC) is a relatively uncommon high-grade malignancy of the salivary gland that may arise de novo or observed in the carcinomatous component of carcinoma ex-pleomorphic adenoma. SDC was originally described as resembling high-grade ductal carcinoma of the breast with cribriform morphology, roman bridge formation, and prominent central necrosis [1,2]. However, variants of SDC such as sarcomatoid, mucin-rich, micropapillary, and oncocytic forms have been described owing to increasing significance on the apocrine phenotype and androgen receptor positivity [3,4,5,6,7]. SDC with rhabdoid features (SDC-RF) is a rare form of salivary gland neoplasm with only 30 patients reported in the worldwide literature [8,9,10,11]. SDC-RF was originally described by Kusafuka et al. in 2013 [12]. It is described as the salivary counterpart of pleomorphic lobular carcinoma of the breast (PLCB) due to the discohesive diffusely infiltrating neoplastic cells with loss of E-cadherin expression and *CDH1* mutations [10,13]. A recent study has suggested that given its divergent finding of discohesive architecture, distinctively aggressive behavior, and striking similarity of the apocrine phenotype and androgen positivity, it is controversial whether SDC-RF should be classified as a variant of the SDC family or a standalone entity [11]. In this report, we describe a case of SDF-RF of the parotid gland with no E-cadherin expression, decreased β-catenin expression, and its immunophenotypic profile.

## 2. Materials and Methods

A 73-year-old male with a significant history of tobacco smoking (100 pack years) presented to the Otolaryngology clinic of University of Tennessee Health Science Center with right facial/neck swelling and intermittent pain of 2 months duration. The patient stated the symptoms had gotten worse over time. There was no facial twitch or weakness. Palpation of the right parotid gland revealed firmness to the tail and inferior aspects of the gland. The patient’s medical history was positive for hypertension, chronic kidney disease, chronic pancreatitis, diabetes mellitus, and pulmonary emphysema. Imaging studies revealed an ill-defined increased density and enhancement of the right parotid gland involving the superficial and deep portions of the gland. There was a persistent loss of delineation within the posterior parotid gland and the adjacent posterior belly of the digastric muscle. An impression of inflammatory changes versus an infiltrating parotid neoplasm was rendered. The patient was prescribed Augmentin 875 mg BID for 10 days to help rule out an infectious/inflammatory process. Two weeks later, the patient returned for a follow-up review with no improvement of symptoms. 

**Figure 1:**
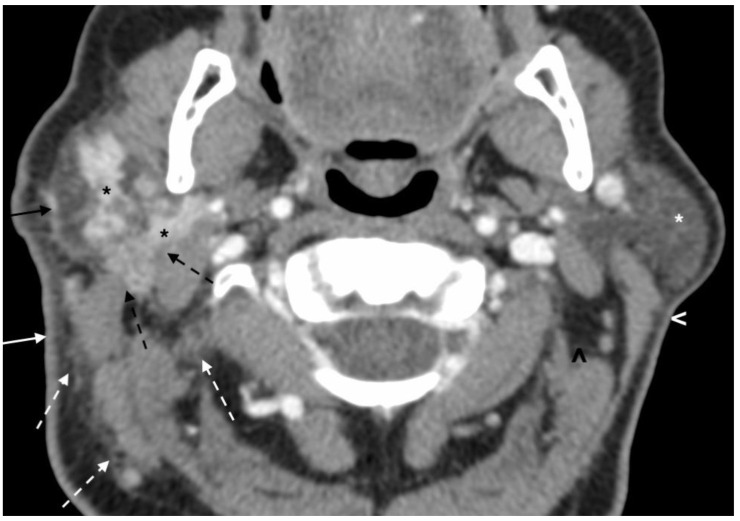
Initial post-contrast CT scan of the neck demonstrates a nodular and infiltrative lesion (black asterisks) centered in the right parotid gland, and significant surrounding fat stranding and fat plane effacement (dashed white arrows) involving the adjacent neck spaces and structures. Both the right sternocleidomastoid and posterior belly digastric muscles have ill-defined margins with the lesion (dashed black arrows). Additionally, there is thickening of the fascial plane (solid black arrow) and overlying skin (solid white arrow). On the contralateral side, there is maintenance of the fat planes (black arrowhead), normal skin (white arrowhead) and fascial plane thickness, and normal CT scan appearance of the parotid gland (white asterisk).

**Figure 2:**
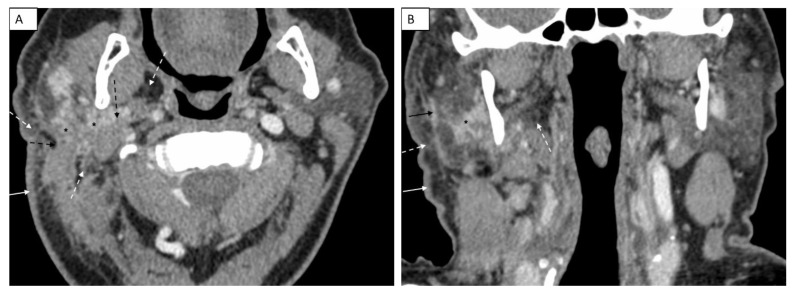
A follow-up post-contrast CT scan of the face 6 weeks later shows worsening skin thickening (white arrow), fat stranding and fat plane effacement (dashed white arrows), ill-defined margins with adjacent sternocleidomastoid and posterior belly digastric muscles with asymmetric enlargement (dashed black arrows) and increased ill-defined margins of the primary lesion in the right parotid gland (black asterisks) on the axial image (**A**). There is infiltration of parapharyngeal fat (dashed white arrow), increased ill-defined margins of the primary lesion in the right parotid gland (black asterisks) and fascial plane thickening (solid black arrow) on the coronal image (**B**).

**Figure 3:**
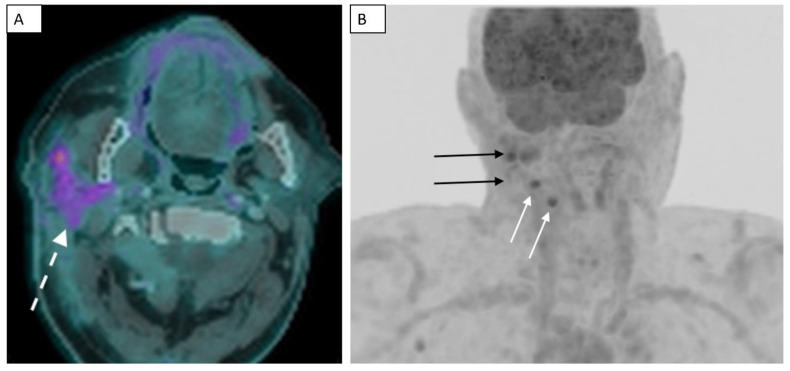
Fused PET–CT scan demonstrates FDG uptake in the infiltrative primary lesion in the right parotid gland (dashed white arrow) (**A**). The 3D MIP image from the PET–CT scan shows the asymmetric ill-defined FDG uptake in the parotid gland and adjacent soft tissues of the right neck (solid black arrows), as well as FDG uptake in two ipsilateral lymph nodes (solid white arrows) (**B**).

The initial post-contrast CT scan of the neck demonstrated a nodular and infiltrative lesion centered in the right parotid gland. There was significant surrounding fat stranding and fat plane effacement involving the adjacent neck spaces and structures. Both the right sternocleidomastoid and posterior belly digastric muscles had ill-defined margins with the lesion. Additionally, there was thickening of the fascial plane and overlying skin (Figure 1). A follow-up post-contrast CT scan of the face 6 weeks later showed worsening skin thickening, fat stranding and fat plane effacement, and ill-defined margins with adjacent sternocleidomastoid and posterior belly digastric muscles with asymmetric enlargement on the axial image (Figure 2A) and coronal image (Figure 2B), suggesting infiltration of these soft tissues with tumor. There was infiltration of parapharyngeal fat and fascial plane thickening (Figure 2B). Despite the patient presenting for PET–CT scan with an elevated blood glucose, which decreased sensitivity of the examination, the fused PET–CT scan image demonstrated FDG uptake in the infiltrative primary lesion in the right parotid gland (Figure 3A). The 3D maximum intensity projection image from the PET–CT scan showed the asymmetric ill-defined FDG uptake in the parotid gland and adjacent soft tissues of the right neck, as well as FDG uptake in two lymph nodes, compatible with infiltrative tumor and nodal metastases, respectively (Figure 3B).

**Figure 4:**
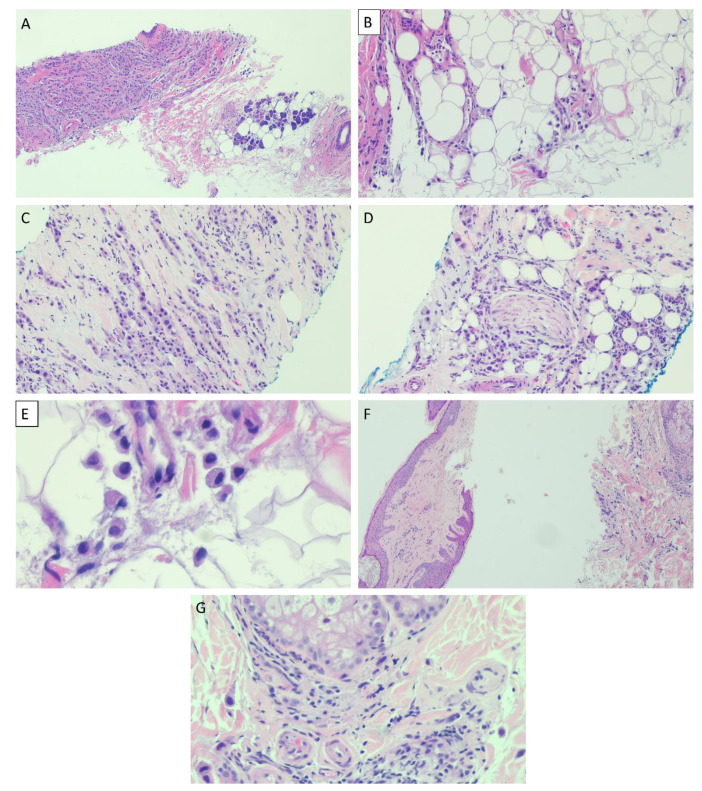
Histopathologic photomicrographs of a salivary duct carcinoma with rhabdoid features. A diffuse infiltrate of tumor cells is seen within parotid gland ((**A**), ×40) and in surrounding adipose tissue ((**B**), ×100). The tumor cells are disposed as single files ((**C**), ×100) and perineural invasion is evident ((**D**), ×100). Single cells infiltrating adipose tissue without supporting stroma, the tumor cells have a plasmacytoid morphology with eccentric nuclei and fine, foamy cytoplasm ((**E**), ×400). Skin biopsy shows infiltration of dermis and skin appendages by single cells with no appreciable stroma accompanying the tumor cells ((**F**), ×40) and ((**G**), ×400).

**Figure 5:**
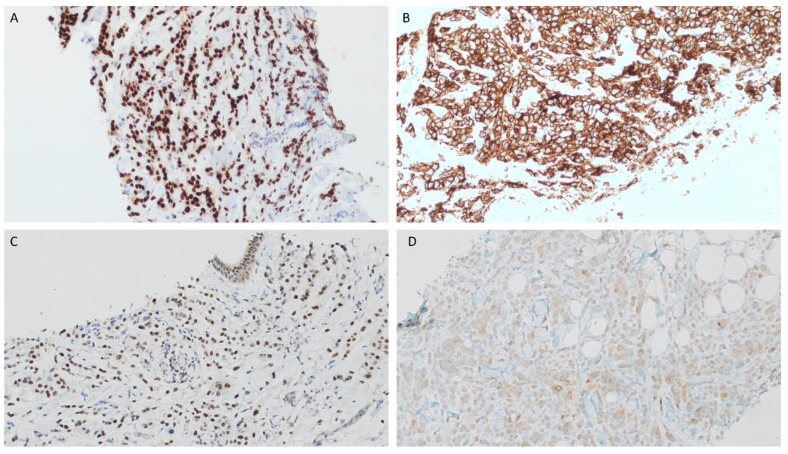
Histopathologic photomicrographs of a salivary duct carcinoma with rhabdoid features. The tumor shows diffuse, strong reactivity with androgen receptor antibody ((**A**), ×200). Complete circumferential membranous staining is identified in tumor cells with HER2 ((**B**), ×200). The tumor cells are positive for GATA3 ((**C**), ×100), and GCDFP shows focal positivity ((**D**), ×200).

**Figure 6:**
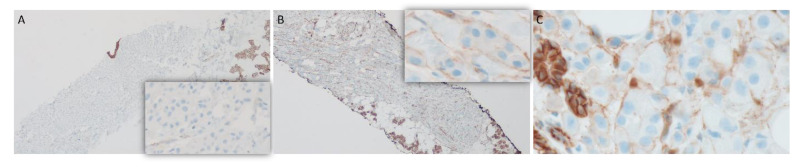
There is complete loss of E-cadherin expression in tumor cells in contrast to strong membranous staining in normal salivary tissue ((**A**), ×40 and inset ×200). Most tumor cells show weak intensity and incomplete membranous staining with β-catenin, in contrast to complete strong membranous staining of normal salivary acini ((**B**), ×40 and inset ×400). Rare tumor cells show nuclear β-catenin expression ((**C**), ×400).

An ultrasound-guided core needle biopsy was performed. Histopathologically, the biopsy showed a diffuse infiltrate of discohesive pleomorphic round cells singly and in single cell files within the parotid gland and surrounding adipose tissue. The stroma ranged from being fibrotic to histologically indiscernible. Typical rhabdoid morphology was not identified. While nuclei were eccentrically placed giving a plasmacytoid appearance, the cytoplasm had a fine, foamy appearance instead of dense eosinophilia of true rhabdoid cells (Figure 4A–F). An extensive panel of immunohistochemical stains was performed. The following stains were positive in the tumor cells: Cytokeratins Oscar and 7, CD138, GATA3, GCDFP, androgen receptor (AR), and HER2 (clone 4B5, Ventana UltraView Universal DAB detection) (Figure 5). Other immunostains were performed to rule out metastasis and were negative: CK20, S100, p40, Melan A, CDX2, TTF1, ER, SATB2, DOG1, synaptophysin, and chromogranin. Additionally, the tumor cells showed complete loss of E-cadherin and decreased membranous β-catenin expression. Rare tumor cells showed nuclear expression of β-catenin (Figure 6). A diagnosis of salivary duct carcinoma was rendered. A few weeks later, the skin of the neck and supraclavicular region appeared infiltrated by tumor causing right neck and shoulder pain and swelling. The skin biopsy from this region showed infiltration of dermis and skin adnexa by the tumor cells (Figure 4G).

The patient’s proposed care was discussed at the Methodist University Hospital Multidisciplinary Head and Neck Tumor Board. The imaging studies were reviewed, including PET–CT images which showed ipsilateral nodal metastases but no distant metastases. Given the patient’s comorbid conditions and involvement of the parapharyngeal space, supraclavicular fat pad, dermis, and skin without a clearly defined surgical target, the tumor was deemed unresectable. The recommendation of the tumor board was for induction systemic treatment with anti-HER2 therapy (Herceptin and Pertuzumab). At the last follow-up, the patient was alive, with complete locoregional control and brain metastases (21 months post-initial presentation). An electronic search was performed in the following registries for papers published up to June 2023: PubMed, Embase, and Web of Science. For the database searches, the keywords searched were “salivary gland”, “salivary duct carcinoma”, and “salivary duct carcinoma with rhabdoid features”.

## 3. Discussion

SDC-RF is a rare form of salivary gland neoplasm with only 30 cases reported in the literature [8,9,10,11]. The tumor is characterized by diffuse infiltrative discohesive ovoid/round eosinophilic cells with pleomorphic nuclei that immunophenotypically express AR and GCDFP, and exhibit loss of/or abnormal E-cadherin expression [8,11]. Our case and previously reported cases suggest that SDC with rhabdoid features may be a misnomer [8,13]. The tumor cells have a plasmacytoid/rhabdoid appearance due to the eccentric placement of nuclei. However, the histologic hallmarks of rhabdoid differentiation such as a dense eosinophilic cytoplasm with paranuclear inclusions and vesicular nuclei with prominent nucleoli are not uniformly identified. Kusafuka et al. confirmed a lack of immunophenotypic features of rhabdoid differentiation in these tumors [8]. Our review of the literature of reported SDC-RF cases reveals that there is a predilection for males (25/30, 83%), parotid gland (21/29, 72%), and patients older than the 6^th^ decade of life (25/30, 83%) [8,9,10,11]. Our reported case does match these tendencies. Immunophenotypically, all SDC-RF cases except one were positive for AR and GCDFP (29/30, 97%), 17/21 (81%) were positive for HER2, and loss or decreased expression of E-cadherin was reported in (28/30, 93%) cases [8,9,10,11]. Alterations in *CDH1* (gene that encodes E-cadherin) have been reported in up to 72–86% of SDC-RF cases [8,11]. The study by Rooper et al. reported a 100% expression of GATA3 in all 7 SDC-RF cases evaluated [14]. GATA3 is a transcription factor that regulates the development of different tissues and GATA3 is expressed in carcinomas of multiple tissue types, such as mammary, urothelial, anal, cervical, lung, and, especially, salivary glands (classic SDC and secretory carcinomas) [15]. Our case shared similar immunophenotypic expressions of AR, GCDPF, HER2, and GATA3 positivity and complete loss of E-cadherin. The clinical and immunophenotypic features of SDC-RF reported in the literature are summarized in Table 1.

E-cadherin is a transmembrane protein that forms homotypic cell–cell adhesion structures called adherens junctions. The intracellular domain binds to catenins, of which β-catenin interacts with the actin cytoskeleton and plays a pivotal role in intracellular signaling promoting cell proliferation and survival [16]. Loss of E-cadherin has been shown to decrease cell adhesion, increase cell motility, and cause alterations in multiple downstream pathways conferring increased dissemination capabilities to breast cancer cells in vitro [17]. Our case showed infiltrating single cells without supporting stroma in adipose tissue and the skin of the neck consistent with decreased cell adhesion and increased motility. In the same report, aberrant cytoplasmic and nuclear expression of functionally active β-catenin was demonstrated in cells with E-cadherin loss. In contrast, all reported cases of E- cadherin loss have shown either a decrease or loss of β-catenin expression. Aberrant cytoplasmic expression was noted in a few cases, but nuclear expression has not been shown [8,10]. Our case showed nuclear expression in rare tumor cells only. While E-cadherin loss is a prototypic feature of invasive lobular carcinoma of breast and diffuse gastric carcinoma, several human tumors are known to lose expression in variant forms [18]. Those tumors with E-cadherin loss in other body sites are usually associated with an aggressive behavior. Therefore, loss of E-cadherin in SDC could potentially be a feature of tumor de-differentiation instead of a distinct salivary tumor subtype [11].

Molecularly, SDC-RF appears to be defined by *CDH1* alteration, mostly mutations and a recently identified *CDH1::CORO7* fusion [8,11]. Other genetic mutations have been reported in SDC-RF, such as mutations in *PIK3CA*, *HRAS*, *TP53*, *PTEN*, and *AKT1* [11]. Mutations in *PIK3CA*, *HRAS*, and *NRAS* have been reported in classic SDC [19,20]. Many salivary gland neoplasms are known to have recurrent genetic alterations: *PLAG1* (fusion partners *CTNNB1*, *LIFR*, *ND4*, *NFIB*, *TGFBR3*, *FGFR1*, and *CHCHD7*) in pleomorphic adenoma (PA), carcinoma ex-PA, and myoepithelial carcinoma [21,22,23,24,25]; *HMGA2* (fusion partners *FHIT*, *NFIB*, *WIF1*, and *TMTC2*) in PA and carcinoma ex-PA [25,26,27]; *MYB/MYBL1::NFIB* in adenoid cystic carcinoma [28,29]; *MAML2::CRTC1/3* in mucoepidermoid carcinoma [30]; *PRKD1/2/3* (fusion partners *ARID1A*, *DDX3X*, *PPP2R2A*, *PRKAR2A*, *SNX9*, *ATL2*, and *STRN3*) in polymorphous adenocarcinoma/cribriform adenocarcinoma of the salivary gland [31,32,33,34,35,36]; *ETV6* (fusion partners *NTRK3*, *RET*, and *MET*) in secretory carcinoma [37,38,39]; *SS18* (fusion partners *MEF2C* and *ZBTB7A*) in microsecretory adenocarcinoma [40]; RET (fusion partners *TRIM27/33* and *NCOA4*) in intraductal carcinoma [41,42]; *HTN3-MSANTD3* and *NR4A3* upregulation in acinic cell carcinoma [43,44,45]; *AKT1* mutations in epithelia–myoepithelial carcinoma, intraductal papillary mucinous neoplasm, and salivary mucinous adenocarcinoma [46,47,48]; *CTNNB1* mutations in basal cell adenoma and basal cell adenocarcinoma [49,50]; and *BRAF* mutations in sialadenoma papilliferum [51,52,53].

The majority of SDC-RF patients were treated with surgery alone or followed by chemoradiation or radiation therapy [8,9,10,11]. Of the 21 patients with clinical outcome information, 60% (12/20) of the patients died of the disease, 35% (7/20) of the patients have no evidence of disease, one patient died of other causes, and one patient was alive with the disease at the last follow-up, suggesting that SDF-RF is a disease with poor prognosis [8,9,10,11]. Our patient was managed with anti-HER2 therapy (Herceptin and Pertuzumab) due to the unresectable nature of the tumor and achieved complete locoregional control. Unfortunately, the patient developed distant brain metastases. Given the poor prognosis associated with the conventional therapeutic approach (surgery +/− chemoradiation) in managing SDC-RF, the use of androgen deprivation therapy (ADT) and anti-HER2 therapies may be more beneficial in the management of HER2 positive/AR positive SDC-RF patients than previously observed. The discohesive diffusely infiltrating nature of the neoplastic cells secondary to loss of E-cadherin expression could make it difficult to obtain a surgical margin free of tumor. The tumor was deemed inoperable given the lack of a defined surgical target. The disease included the parotid, parapharyngeal fat, the supraclavicular fat pad, skin, and dermis down to level V and extending to the deep muscles of the neck. This spread was not contiguous, and based on imaging, was spreading in a lacey pattern as opposed to a discrete mass. Resection would have involved a radical parotidectomy, resection of the parapharyngeal space, and modified radical neck dissection with resection of the skin of the neck extending from the face, over the trapezius and down to the clavicle. This was deemed overly morbid, especially given the surgical team’s assessment that we were unlikely to obtain negative surgical margins, and consequently the patient would still need adjuvant therapy. Studies have shown that SDC patients managed with ADT or anti-HER2 therapies have a better prognosis compared to SDC patients managed with conventional therapy [54,55,56]. A recent study reported that locally advanced, recurrent, or metastatic SDC patients managed with anti-HER2 therapy had significantly better prognosis compared to the patients managed with ADT [56].

## 4. Conclusions

We report a case of salivary duct carcinoma with “rhabdoid” features of the parotid gland with no E-cadherin expression. The tumor’s lack of cohesion and diffuse infiltrating pattern of spread throughout the neck region rendered it surgically unresectable. The patient was managed with anti-HER2 therapy (Herceptin and Pertuzumab), alive with complete locoregional control and distant brain metastases (21 months post-initial presentation).

## Figures and Tables

**Table 1:** Clinical and immunophenotypic features of salivary duct carcinoma with rhabdoid features reported in the literature.

Author	Age	Sex	Site	AR	E-Cadherin	GCDFP	HER2	Treatment	Follow-Up	Outcome
Kusafuka et al. [8]	44	M	Parotid	+	Total Loss	+	+	Surgery, RT	21	DOD
	66	M	SMG	+	D Cyt	+	−	Surgery	26	DOD
	39	M	Parotid	+	Total Loss	+	+	Surgery, CRT	15	NED
	73	M	Parotid	+	D	+	+	Surgery	NA	LTFU
	75	M	Parotid	+	Total Loss	+	+	RT	NA	DOD
	36	M	Parotid	+	D	+	+	Surgery	78	NED
	83	M	SMG	+	Total Loss	+	−	Surgery	NA	LTFU
	85	F	Parotid	+	D	+	+	Surgery, RT	NA	DOD
	81	M	SMG	+	D	+	+	Surgery	NA	DOD
	68	F	Parotid	+	D	+	+	Surgery	NA	NED
	78	F	SMG	+	Total Loss	+	+	Surgery	NA	NED
	71	M	Unknown	+	D Cyt	+	+	Surgery	NA	DOC
	54	M	SMG	+	Total Loss	+	−	Surgery	NA	DOD
	82	M	Parotid	−	Total Loss	−	+	Surgery	NA	DOD
	81	M	Parotid	+	Total Loss	+	+	Surgery, RT	NA	LTFU
	63	F	Parotid	+	Total Loss	+	+	Surgery, RT	NA	NED
	62	M	Parotid	+	Total Loss	+	+	Surgery, CRT	NA	AWD
Otsuru et al. [9]	82	M	SMG	+	ND	+	+	Surgery, CRT	12	DOD
Akaki et al. [10]	60	M	Parotid	+	D	+	−	Surgery	79	LTFU
	82	M	Parotid	+	D	+	+	Surgery	60	LTFU
	76	M	Parotid	+	D	+	+	CRT	10	DOD
Rooper et al. [11]	65	M	Parotid	+	Total Loss	+	NA	Surgery, CRT	48	DOD
	63	M	Parotid	+	Total Loss	+	NA	Surgery, CRT	20	DOD
	78	M	Parotid	+	Total Loss	+	NA	Surgery, CRT	5	NED
	66	M	Parotid	+	D Cyt	+	NA	Surgery, CRT	10	DOD
	75	M	Parotid	+	+	+	NA	NA	NA	LTFU
	83	M	Parotid	+	Total Loss	+	NA	NA	NA	LTFU
	67	M	Parotid	+	+	+	NA	NA	NA	LTFU
	67	M	SMG	+	Total Loss	+	NA	Surgery, CRT	6	NED
	75	F	Buccal	+	Total Loss	+	NA	NA	NA	LTFU
This case	73	M	Parotid	+	Total Loss	+	+	Anti-Her2	21	Locoregional control

M—Male, F—Female, AR—Androgen receptor, GCDFP—Gross cystic disease fluid protein, HER2—Human epidermal growth factor receptor 2, D—Decreased, D Cyt—Decreased with cytoplasmic expression, NA—Not available, CRT—Chemoradiation therapy, DOD—Died of disease, LTFU—Lost to follow up, NED—No evidence of disease, AWD—Alive with disease.

## Data Availability

The data are unavailable due to privacy or ethical restrictions. Please contact the corresponding author.
